# Root systems analysis branches out

**DOI:** 10.1038/msb.2013.51

**Published:** 2013-10-22

**Authors:** Klaus Palme, William Teale

**Affiliations:** 1 Faculty of Biology, Institute of Biology II/Botany, Albert-Ludwigs-University of Freiburg, Freiburg, Germany; 2 Centre of Biological Systems Analysis, Albert-Ludwigs-University of Freiburg, Freiburg, Germany; 3 Freiburg Institute of Advanced Sciences (FRIAS), Albert-Ludwigs-University of Freiburg, Freiburg, Germany; 4 Centre for Biological Signalling Studies (BIOSS), Albert-Ludwigs-University of Freiburg, Freiburg, Germany

Land plants secure a constant supply of water and nutrients by developing a deep and densely branched root system. Nutrients are often distributed unevenly in the soil; formation of lateral roots must therefore be responsive to environmental conditions. Over time, lateral root initiation has become an accessible model system for studying the basic regulatory mechanisms that control postembryonic organ development. While there has been much interest in the phenomenology of root branching, it is only recently that the underlying molecular processes have started to be understood (Malamy, 2005).

The recent article by Bennett and colleagues (Péret et al, 2013) presents an exciting example of how an integrated experimental and mathematical approach can elucidate the signaling pathways that drive lateral root emergence. The authors chose their model plant wisely: *Arabidopsis thaliana* has a relatively simple root anatomy when compared to other plants such as grasses. Arabidopsis roots are composed of single concentric layers of epidermal, cortical, endodermal and pericycle cells, which surround a double-stranded vascular cylinder (Figure 1). Lateral roots originate from a subset of pericycle cells, called founder cells, adjacent to each xylem strand. The most important determinant of lateral root initiation is auxin. Oscillating waves of auxin, driven by auxin transport proteins in the protoxylem, mark lateral root founder cells (Ditengou et al, 2008; Moreno-Risueno et al, 2010). The formation of the lateral root primordium then proceeds through a well-defined program of periclinal (parallel) and anticlinal (perpendicular) divisions. Crucially, lateral root primordia may then have to emerge through up to 15 overlying tissue layers (depending on the plant species in question). As well as initiating primordium development, auxin transport, this time via its influx carrier LAX3, was identified as key regulator of lateral root emergence (Swarup et al, 2008). Specifically, auxin acts as a local signal, which induces the expression of LAX3 in those cortical and epidermal cells which lie directly over the new lateral root primordium. In these cells, LAX3 reinforces the auxin-dependent induction of a selection of cell-wall-remodeling enzymes, including polygalacturonase, which promote cell separation and tissue softening.

The involvement of LAX3 nevertheless left an open question: how is a tissue-scale cell-to-cell coordination achieved if the signal (auxin) is being cleared from the tissue by its specific cellular uptake carrier? Péret et al (2013) addressed this question by elegantly integrating experimental approaches such as genetic manipulation and imaging of fluorescent reporters with mathematical modeling using realistic three-dimensional cell and tissue geometries. Analysis of an initial version of the model, considering the regulation of LAX3 expression by auxin alone, allowed Péret et al (2013) effectively to test *in silico* whether this mechanism was sufficient to explain the observed expression pattern. These simulations predicted a much higher variation in the number and position of LAX3-expressing cells than was observed experimentally (Figure 1A), suggesting that an important regulatory element was missing from this initial model. However, when the model was extended by including the expression of an auxin efflux carrier, the discrepancy was resolved. Auxin was able to move from one cell to another, and the all-important component of cell-to-cell communication was described (Figure 1B). Indeed, sequential induction of the PIN3 efflux carrier followed by expression of the LAX3 influx carrier allowed the model to produce a coordinated sharpening of the border of LAX3 domains. In addition, these domains were robust to variations in the size of the original auxin source, which result from natural variations in root geometry.

The study by Péret et al (2013) provides important insights into the dynamics of the developmental process of lateral root emergence, but many challenges lie ahead. Although computational modeling of auxin transport has been increasingly popular in recent years (Santos et al. 2010), the limitations of such simulations have become increasingly clear, as ‘realistic parameters', short-hand in most cases for ‘currently unmeasurable rates and concentrations', are used as their basic building blocks. Truly quantitative models are so far lacking. For instance, as it stands, auxin flux rates within cells and tissues cannot be directly determined, and proteins cannot be quantified *in vivo* at cellular and subcellular resolutions. The continuous emergence of novel technologies, such as light sheet microscopy and sensors for quantitative ratiometric time-resolved analysis of auxin dynamics (Brunoud et al, 2012; Krzic et al, 2012; Tomer et al, 2012), may allow us to overcome these limitations in the near future.

## Figures and Tables

**Figure 1:**
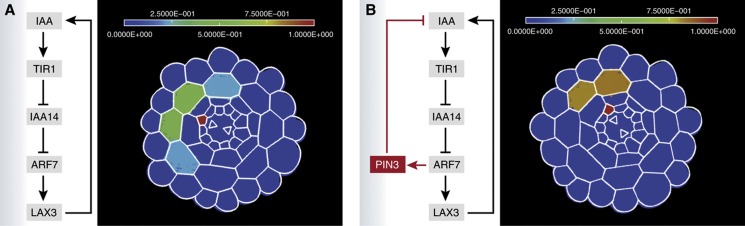
Interplay between auxin efflux and influx carriers is required for correct and robust patterning. (**A**) Regulation of LAX3 expression by auxin alone leads to the expression of LAX3 in 3–4 cortical cell files, which does not correspond to experimental observations. (**B**) It is only when the auxin efflux carrier PIN3 is included in the model that the correct LAX3 expression pattern can be obtained, resulting in robust expression restricted to two cortical cell files, thereby delimiting cell separation in advance of new lateral root primordia. Abbreviations: IAA, Auxin; TIR1, Auxin receptor component; IAA14, Aux/IAA protein 14; ARF7, Auxin response factor 7; PIN3, Auxin efflux transporter; LAX3, Auxin influx transporter.
